# Implication of cystic fluid cytology of renal cell carcinoma on surgical practice

**DOI:** 10.1186/s12894-022-01144-y

**Published:** 2022-11-25

**Authors:** Kyung Jae Hur, Qais Hooti, Dongho Shin, Yong Hyun Park, Woong Jin Bae, Hyukjin Cho, U-syn Ha, Ji Youl Lee, Yeong Jin Choi, Sung-Hoo Hong

**Affiliations:** 1 Department of Urology, Seoul St. Mary’s Hospital, College of Medicine, The Catholic University of Korea, 222, Banpo-Daero, Seocho-Gu, Seoul, 06591 Republic of Korea; 2 Department of Urology, Daegu Fatima Hospital, Daegu, South Korea; 3 Department of Urology, The Royal Hospital, Muscat, Sultanate of Oman; 4 Department of Pathology, Seoul St. Mary’s Hospital, College of Medicine, The Catholic University of Korea, Seoul, Republic of Korea

**Keywords:** Renal cell carcinoma, Cystic fluid cytology, Bosniak grade, Cyst rupture

## Abstract

**Objectives:**

To evaluate the incidence of positive cystic fluid cytology and its risk factors in cystic renal cell carcinoma (RCC) addressing its implication on the current surgical practice.

**Methods:**

All clinically diagnosed Bosniak III, IV cystic renal masses from March 2019 to August 2022 were studied prospectively. Database of patients’ demographics and cystic tumor characteristics were recorded. Partial or radical nephrectomies were performed by either laparoscopic or robotic approach. Cystic fluid was collected right after specimen retrieval in the surgical field and examined by pathologist. Cytology results were compared to the demographic, perioperative variables using univariate and multivariate analysis.

**Results:**

A total of 70 patients of histologically confirmed cystic RCC were included. Sixty seven patients underwent radical nephrectomy with laparoscopic or robotic approaches, while 3 patients underwent radical nephrectomy. There was no intraoperative cystic rupture or fluid spillage. Positive cystic fluid cytology findings were identified in 34 (48.6%) patients, while negative cystic fluid cytology were identified in 36 (51.4%) cases. Definite malignant cells were observed in 28 patients while the other six patients showed highly suspicious atypical cells. Histologically, 24 (70.8%) patients were proven clear cell RCC and 25 (73%) showed Fuhrman grade 1 or 2 in final histologic review in positive group. Univariate and multivariate regression analysis between positive and negative cytology groups showed that the presence of the malignant cells in cystic fluid was significantly associated with patients’ age (> 55 years) and Bosniak grade of cystic tumor (*p* < 0.05).

**Conclusions:**

Definite malignant cells in cystic fluid cytology were observed through our study. Additionally, patients’ age (> 55 years) and Bosniak grade were the significant risk factors of positive cytology in cystic RCC. Therefore, necessity of meticulous manipulation of cystic renal tumors, despite their clinical features, should not be underemphasized to avoid the least possible tumor cell seeding in case of cystic rupture when operating such high risk of positive cytology.

## Introduction

Cystic renal tumors comprise 5–10% of all renal cell carcinomas (RCC) [1–3]. The risk of fluid spillage and seeding of malignant cells due to cyst rupture is one of the major concerns while operating cystic renal tumors [4–7]. Occurrence of intraoperative rupture of cystic RCC has been addressed in the previous literatures [4, 8, 9].

In retrospective analysis, Pradere et al. reported intraoperative ruptured cystic RCC in 18.7% of 268 patients. None of those ruptured cases had local recurrence or distant metastasis on long-term follow up [8]. Furthermore, laparoscopic fine needle aspiration (FNA) and cystic wall biopsy of cystic RCC did not show any significant oncological consequences [10]. Hayakawa et al. reported that preoperative cystic fluid assessment has shown a positive malignant cytology in only 9–14% of subsequently proved RCC [10, 11].

However, if clinical practice is based on these previous reports, urologists could be less careful during operation of cystic portion of RCC. Consequentially, risk of cystic rupture and the least possible tumor cell seeding could be underestimated. Furthermore, previously published reports were based on preoperative FNA study, which have limitations of inadequate sampling and high false negative rates. Actual presence of malignant cell in cystic fluid and its incidence was not fully evaluated in these previous studies.

Therefore, we prospectively investigated the incidence of positive and negative cystic fluid cytology of histologically confirmed cystic RCC and its risk factors. Our purpose is to precisely access the actual presence of malignant cells in the cystic fluid of RCC by performing direct cystic fluid aspiration from the retrieved specimen in the surgical field, emphasizing its oncological implication on the current clinical practice.

## Materials and methods

### Patient selection

All cystic renal tumors diagnosed by abdominal computed tomography (CT) scan were evaluated. Demographic data, cystic tumor size, clinical stage and Bosniak category were collected, along with postoperative histologic and cytological results. All patients were evaluated with clinical examination, laboratory test including complete blood cell count, liver, renal function test and electrolyte profiles, chest and abdominal CT scan at periodic schedule with follow up period for 6–32 months.

### Inclusion and exclusion criteria

The patients included were those with Bosniak category III and IV on preoperative CT scan. Cystic degeneration tumors were excluded. Cases of insufficient volume of cystic fluid sampling (less than 5 ml) or benign histology found at final pathology were excluded. Cases of polycystic kidney disease were also excluded.

### Surgical methods

All surgeries were performed by single high volume surgeon. Surgical methods included laparoscopic partial nephrectomy (LPN), laparoscopic radical nephrectomy (LRN), and robot-assisted partial nephrectomy (RAPN).

### Cystic fluid cytology analysis

After specimen retrieval, cystic fluid was aspirated directly from the cysts using 14 Gauge needle in the surgical field. The maximum possible sample volume was obtained. Sample was sent to pathology laboratory to be centrifuged for 5 min, and supernatant liquid was discarded. Cell pellet and washing solution were mixed, and 10 min of second centrifugation was done. After centrifugation, cell pellet and 20 ml washing solution were mixed to make a slide preparation. Prepared slides were stained with Papanicolaou smear. Pathologists who were specialized in urologic specimen analysis performed the cytological study under high power field light microscope.

Cytological diagnosis was classified into 3 categories of definite malignant cells, atypical cells and negative for malignant cells. Positive cytology was defined as the presence of definitive malignant cells or suspicious atypical cells.

### Statistical analysis

The data were analyzed using IBM SPSS Statistics ver. 24.0 (IBM Co., Armonk, New York, USA). Demographic and perioperative data of each group were compared using paired t-test. Pearson's chi-square test was used for comparative analysis of two independent cytology group. Univariate and multivariate linear regression analysis were used to determine influential factors of positive cystic fluid cytology. *p* value < 0.05 was considered to be statistically significant.

### Ethics declarations

This study was approved by the Institutional Review Board of Seoul St. Mary’s Hospital, the Catholic University of Korea, College of Medicine (Approval number: KC20RISI0357), and is in accordance with Helsinki declaration and its later amendments. Written informed consent was obtained from all patients.

## Results

A total of 548 RCC patients underwent surgical resection from March 2019 to August 2022 in our institution. Among them, 74 patients were radiologically diagnosed as cystic renal tumors. Two cases had insufficient fluid sampling, while the other two cases were found to be benign simple cyst or oncocytoma at final histology.

Among the final 70 enrolled patients, 43 were males and 27 were females. Patients’ demographics and tumor characteristics of both positive and negative cystic fluid cytology cases are presented in Table 1. Positive cytology was identified in 34 (48.6%) patients. In positive cytology group, cystic mass size ranged from 1.1 to 14 cm with a median of 3 cm in positive cytology group. Bosniak category III cystic lesions were shown in 9 patients (26.5%) while 25 patients (73.5%) were shown category IV.

**Table 1:** Patients’ demographics and tumor characteristics of cystic renal cell carcinoma cytology (total number = 70)

Variables	Positive cytology (n = 34)	Negative cytology (n = 36)	*p* value
Age (years)	56.3 ± 2.11	49.6 ± 2.36	0.037*
BMI (kg/m^2^)	25.1 ± 5.3	26.4 ± 2.3	0.426
Gender			0.59
Male	22 (64.7%)	21 (58.3%)	
Female	12 (35.3%)	15 (41.7%)	
Tumor size (cm)	3.5 ± 0.41	3.81 ± 0.4	0.588
Median tumor size (cm)	3.0	3.3	
Laterality			
Right	16 (47%)	23 (63.8%)	
Left	18 (53%)	13 (36.2%)	
Operation type			
RAPN	26 (76.5%)	30 (83.3%)	
LPN	6 (17.7%)	5 (13.8%)	
LRN	2 (5.8%)	1 (2.9%)	
Bosniak classification			0.002*
III	9 (26.5%)	20 (55.6%)	
IV	25 (73.5%)	16 (44.4%)	
Clinical stage			0.56
cT1a	20 (58.8%)	24 (66.6%)	
cT1b	11 (32.4%)	10 (27.7%)	
cT2a	3 (8.8%)	2 (5.7%)	
Histology subtype			0.084
Clear cell	24 (70.8%)	26 (72.2%)	
Papillary	4 (11.7%)	6 (16.6%)	
Collecting duct	3 (8.8%)	1 (2.9%)	
Chromophobe	2 (5.8%)	3 (8.3%)	
MiT family Xp11.2 translocation	1 (2.9%)	0	
Histology grades			0.14
Grade 1	11 (32.4%)	10 (27.7%)	
Grade 2	14 (41.1%)	18 (50%)	
Grade 3	9 (26.5%)	8 (22.3%)	

Values are presented as mean ± standard deviation, or number (percentage)

BMI, body mass index; RAPN, robot-assisted partial nephrectomy; LPN, laparoscopic partial nephrectomy; LRN, laparoscopic radical nephrectomy

*p < 0.05

**Fig. 1:**
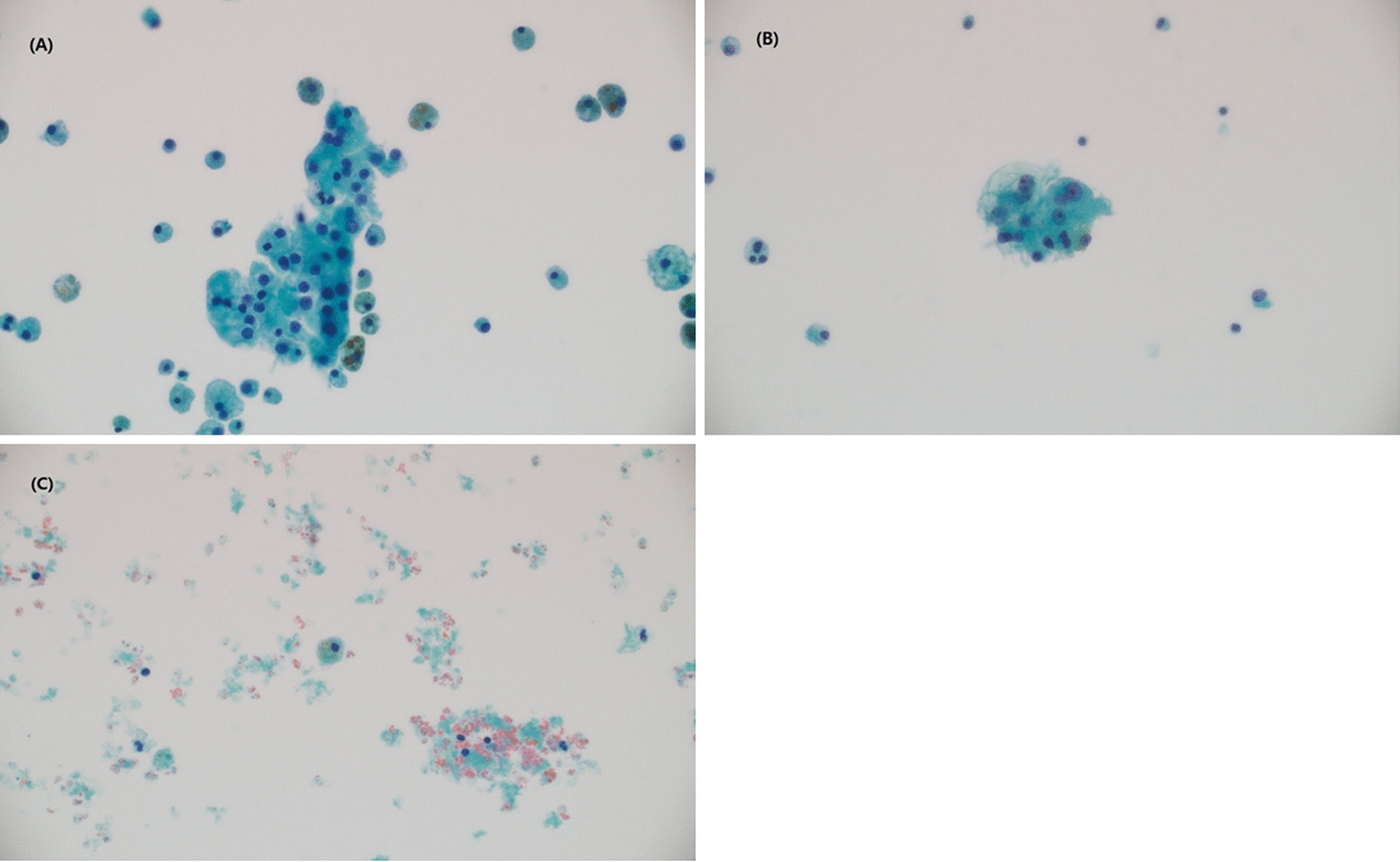
Cytology slides of the cystic fluid. **A** Malignant cells, showing prominent and large hyperchromatic nuclei and large abundant cytoplasm which is densely packed. **B** Atypical cells, showing as dysmorphic nucleus with irregular cell contour, and relatively high ratio of nucleus to cytoplasm (N/C). **C** Negative of malignant cell with macrophages and lymphocytes

Malignant cells showed large hyperchromatic nuclei and prominent nucleoli with large abundant cytoplasm which could be clear vacuolated or densely packed **(**Fig. 1A). Atypical cells were characterized by dysmorphic nucleus and irregular contour, showing relatively high ratio of nucleus to cytoplasm (Fig. 1B). Both cells appeared either in clusters or as a couple of scattered single cells of less than 5 cells/HPF in the histological slide. Negative cytology showed only clusters of macrophages and lymphocytes (Fig. 1C).

All tumors were operated by minimally invasive surgery. Overall, RAPN and LPN were performed in 56 and 11 patients, respectively. Three patients underwent radical nephrectomy due to central location of tumor. Neither cystic rupture nor fluid leakage occurred during surgery or specimen retrieval.

**Fig. 2:**
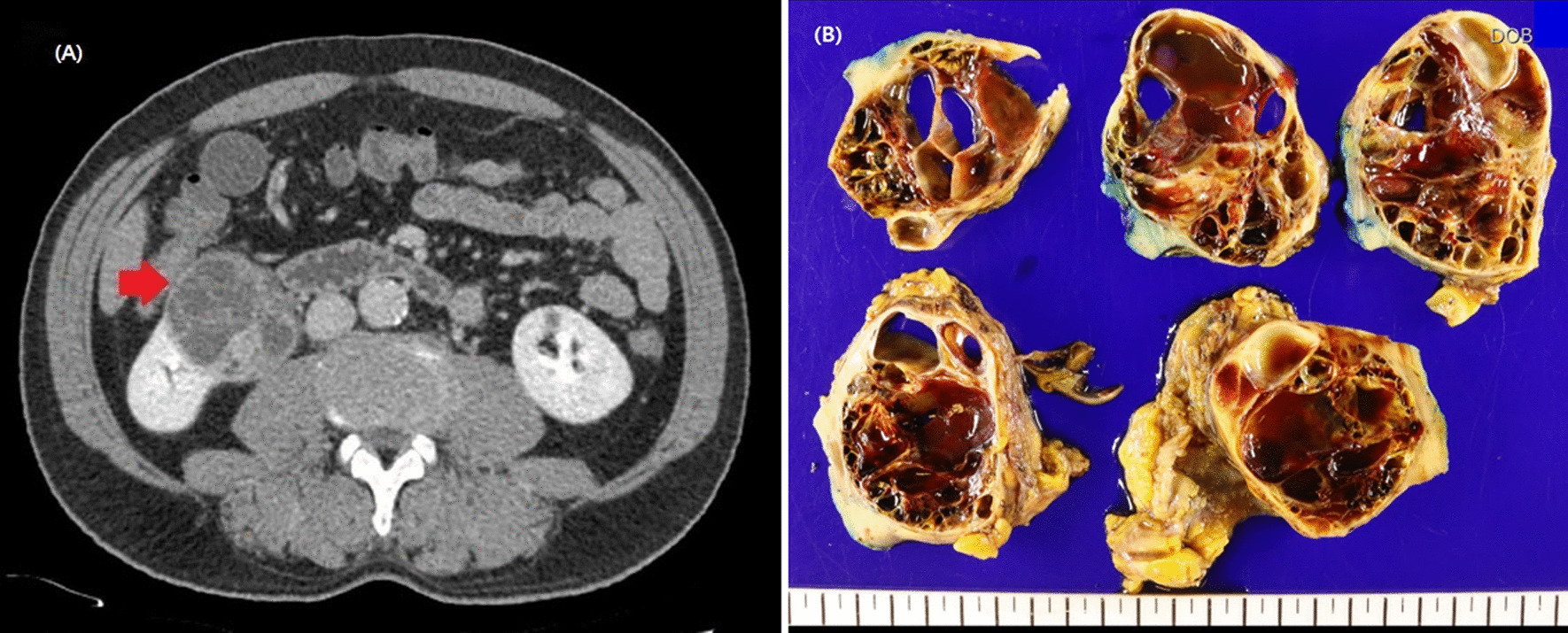
Preoperative contract CT scans of cystic renal tumor and gross picture of retrieved cystic renal cell carcinoma. **A** Bosniak IV renal cyst showing multi-septated cystic mass with enhancing nodular walls. **B** Bosniak IV renal cyst showing gross nodular lesion with multiple septation

Gross pathological assessment of the resected masses confirmed the cystic nature of the tumor shown in preoperative CT scan (Fig. 2). In positive cytology group, histological examination revealed clear cell RCC in 24 (70.8%) patients, type 2 papillary variant in 4 (11.7%) patients and collecting duct carcinoma in 3 (8.8%) patients. One case of MIT family (Xp11.2) translocation type RCC and two cases of chromophobe type RCC were identified.

All tumors were graded histologically using Fuhrman grading system. In positive cytology group, grade 1, 2 and 3 were seen in 11 (32.4%), 14 (41.1%) and 9 (26.5%) cases, respectively. None of the patients had grade 4 diseases. Of the 34 positive cytology tumors, 20 (58.8%) cases were within 4 cm in size (cT1a stage), while 11 (32.4%) were between 4 and 7 cm (staged as cT1b). The negative fluid cytology cysts of the 36 patients showed absence of malignant or atypical cells, but only presence of microscopic macrophages and lymphocytes.

Univariate regression analysis between positive and negative cytology groups showed that the presence of malignant cells in cystic fluid was significantly associated with patients’ age (> 55 years) (*p* = 0.004) and Bosniak grade of cystic tumor (*p* = 0.001). However, patients’ sex (*p* = 0.403) and tumor size (*p* = 0.742) were not significantly associated with incidence of positive cytology in univariate regression analysis. Also in multivariate regression analysis, patients’ age (> 55 years) (*p* = 0.035) and Bosniak grade of cystic tumor (*p* = 0.006) were the significant risk factors of positive cytology. Patients’ sex (*p* = 0.98) and tumor size (*p* = 0.777) were not significantly associated with incidence of positive cytology in multivariate regression analysis (Tables 2, 3). There was no evidence of local recurrence or distant metastasis during the postoperative follow up period.

**Table 2:** Univariate analysis of risk factors associated with positive and negative cystic fluid cytology groups

	R^2^	β	t	95% CI	*p* value
Age (> 55 years)	0.117	0.342	3.0	0.114–0.569	0.004*
Gender (male)	0.01	− 0.102	− 0.842	− 0.351–0.143	0.403
Tumor size (> 4 cm)	0.002	− 0.04	− 0.33	− 0.059–0.042	0.742
Bosniak grade	0.157	0.397	3.563	0.15–0.533	0.001*

CI, confidence interval; β = standard coefficient

*p < 0.05

**Table 3:** Multivariate analysis of risk factors associated with positive and negative cystic fluid cytology groups

	OR	β	t	95% CI	*p* value
Age (> 55 years)	1.23	0.249	2.157	0.018–0.48	0.035*
Gender (male)	0.642	− 0.003	− 0.026	− 0.235–0.229	0.98
Tumor size (> 4 cm)	0.46	0.033	0.284	− 0.041–0.055	0.777
Bosniak grade	3.64	0.336	2.828	0.085–0.494	0.006*

OR, odds ratio; CI, confidence interval (R^2^ = 0.218, β = standard coefficient, *p* = 0.003)

*p < 0.05

## Discussion

Cystic RCC represents about 5–10% of all renal cell carcinomas [1–3]. Tumor violation and cancer cell spillage are the major concerns during surgery, particularly when cystic renal tumors are operated. Urologists may face anxiety if intraoperative cystic RCC rupture is encountered. The stress due to possible tumor cell seeding can adversely affect the surgical and oncological outcomes. However, Pradere et al. reported no local recurrence or distant metastasis on long-term follow up in intraoperative cyst rupture of cystic RCC in 18.7% of 268 patients [8]. Tumorous effect of cystic fluid rupture in cystic RCC has been still on debate, according to previous published reports.

Laparoscopic evaluation with cystic wall biopsy and fluid sampling of 57 indeterminate renal cysts was performed by Limb et al. [10]. Eleven patients were diagnosed to have RCC, out of which only one (9%) had positive cystic fluid cytology. There was no peritoneal or port site recurrence on follow up period. Nephron sparing surgery by laparoscopic or robotic approaches for complex renal cysts is safe, feasible and not inferior to open surgery for solid renal masses [12–15].

Safety of cystic wall puncture for cytology and biopsy during tumor ablation also has been reported in the literature [11–14]. So far, only few cases of needle tract seeding after percutaneous needle aspiration of renal tumors are reported [16, 17]. However, fine needle aspiration cytology (FNAC) of cystic renal tumor is of limited usefulness, probably due to inadequate sampling and false negative results with low accuracy [18, 19]. Hayakawa and colleagues studied FNAC of renal cystic tumors. Positive cytology was identified in only 14% among total of 37 subsequently proved cystic RCC patients [11]. Meanwhile, the risk of cyst rupture associated with intraoperative manipulations should not be neglected because cyst rupture and subsequent fluid spillage might increase risk of local recurrence [13].

Li et al. have reported 10 positive percutaneous FNAC (48%) of 21 documented RCC after surgery. Among those 11 positive cytology cases, there were 4 cases of suspected malignant and 7 cases of clearly malignant cells [20]. One patient had false positive result of histology proven benign cyst.

However, these previous studies have limitations because they were based on preoperative FNA study. FNAC of cystic renal tumor has limitations due to its inadequate sampling and high false negative rates. Consequentially, risk of cystic rupture and the least possible consequential tumor cell seeding could be underestimated. Up to now, detailed evaluation about the actual presence of malignant cell in cystic fluid and the associated risk factor is lacking. Therefore, we prospectively investigated the cystic fluid cytology of histologically confirmed cystic RCC by performing direct cystic fluid aspiration from the retrieved specimen in the surgical field, to figure out the incidence and associated risk factors of malignant cells in the cystic fluid of RCC.

In our data, cystic fluid cytology was positive in nearly half (48.6%) of among total of 70 cystic RCC patients including various histologic subtypes, with clear cell type most common. To the best of our knowledge, this is the highest incidence of positive cystic RCC cytology compared with the previous published literatures. It may be attributed to the prospective analysis associated with accurate sampling from the specimen in the surgical field, and meticulous handling without intraoperative cyst rupture. Also, study of cystic fluid cytology in our study revealed definite existence of cancer cells in cystic fluid using light microscope, which warrants meticulous dissection during surgery of cystic RCC to avoid tumor cell seeding caused by cystic rupture. To overcome the hurdles and limitations of inadequate sampling in CT or ultrasonography guided FNAC, we conducted direct cystic fluid aspiration from the delivered specimen in the surgical field. Furthermore, risk factors of positive cystic fluid cytology have been evaluated through retrieved specimens without cystic rupture.

FNAC may be helpful in diagnosis of RCC in preoperative imaging study. However, the diagnostic accuracy of FNAC in cystic RCC is low and the risk of cystic rupture or fluid leakage exist. According to the results of our study, the presence of tumor cells in the cystic fluid was confirmed in about half of the cases. Hence, preoperative FNAC would better be performed limitedly to selected patients.

Of the 34 positive cytology cases, definite malignant cells were identified in 28 patients while the other six cases showed highly suspicious atypical cells. We included atypical cells in the same group of positive malignant cells because they were assumed to exhibit similar tumorous characteristics with malignant cells, due to speculation of their cell components showing dysmorphic nucleus and high nucleus to cytoplasmic ratio. However, actual evaluation of behavior of these cells was limited due to absence of cystic rupture cases.

We presumed that positive cystic cytology would be associated with patient's old age (> 55 years), Bosniak grade, tumor size (> 4 cm in diameter, cT1a between cT1b) or histological grade of the tumor. Among those variables, patients’ age and Bosniak grade were found to be the significant risk factors of positive cytology. Thirty-one cases (91%) of positive cytology tumors were less than 7 cm in size (clinical stage T1). Clear cell carcinoma was most common histological subtype (24 patients; 70.8%). Both of papillary type 2 variant histology cases were positive of malignant cells. More than two third of these positive cytology tumors were of low Fuhrman Grade 1 or 2. These parameters of positive cytology patients were almost similar to the total patients included in the study.

The results of our data showed that old age and higher Bosniak grade remained as the significant risk factors of positive cytology in cystic RCC. On the other hand, small tumors of Bosniak class III could still harbor malignant or atypical cells in their cystic fluid.

Although tumor cell seeding of ruptured cystic renal cancer is known to be uncommon in previous published studies, our results still warrant that the necessity of meticulous manipulation of cystic renal tumor should not be underemphasized to avoid cystic rupture in older age patients (> 55 years) and higher Bosniak grade (III, IV). The presence of malignant cells in cystic fluid of RCC could be the evidence that warrants the least possible tumor cell implantation in case of cystic rupture.

Obviously, rupture of cystic component of RCC may lead to spillage of tumor cells in the surgical field. However, the evidence regarding ability of these cells to implant and grow is uncertain yet. This definitely necessitates further studies to understand the biology of this type of tumor cells. Detailed analysis of cystic fluid of renal tumors to understand the biological nature and behavior of the tumor cells is important. Different molecular biomarkers like proteins, interleukines, tumor necrosis and growth factors were observed in the cystic fluid [21, 22]. However, clinical significance of the molecular assay particularly when the malignant cells are absent in the cystic fluid, may needs to be evaluated in detail.

Chen et al. compared prognosis of patients with intraoperative cystic ruptures group and the group without cyst ruptures among total of 174 patients, through the evaluation of risk factors of intraoperative cystic rupture [23]. There were 27 (15.5%) intraoperative cyst ruptures. The median follow-up time was 60 months. They reported that 5-year recurrence free survival and cancer free survival in patients with cyst rupture were worse than those without cyst rupture. However, there was no significant difference in overall survival between the two groups. This could be another evidence of tumorous effect of positive cystic fluid cytology when cystic rupture occurred during surgery of cystic RCC.

Several limitations are present in our study. First, follow up period is median-term and numbers of enrolled cases were small. Second, we did not evaluate the cytology of benign renal cysts. It would be helpful to assess the false positive cytology rate to predict the value of FNAC of cystic renal tumors. Also, most common histology of our enrolled cases were clear cell type, but a few of other histologic types including papillary type and other cell types are included. As these tumors have different clinical, pathological and genetic features, further studies regarding correlation between cytology findings and each histologic types will be required. In addition, due to surgeon’s effort and carefulness of not trying to rupture the cystic component of tumor, there was no case of cystic rupture. Paradoxically, actual evaluation of behavior and aggressiveness of these cystic fluid tumor cells was limited due to absence of cystic rupture case. Studying the cystic fluid cytology of simple renal cysts and clarified evaluation of each histologic types of cystic RCC cytology would be required with larger numbers of cases.

## Conclusions

In the present study, patients’ age (> 55 years) and higher Bosniak grade (IV) were found to be the significant risk factors of positive cytology in cystic RCC. Therefore, the necessity of meticulous manipulation of cystic renal tumors should not be underemphasized to avoid cystic rupture and the least possible tumor cell seeding in such high risks of cystic RCC cases. To better understand the cystic fluid cells' ability to implant and grow, further cell studies and culture under similar condition and pressure are recommended.

## Data Availability

The data that support the findings of this study are strictly available from the corresponding author on reasonable request.

## Abbreviations

RCC: Renal cell carcinoma
FNAC: Fine needle aspiration cytology
LPN: Laparoscopic partial nephrectomy
LRN: Laparoscopic radical nephrectomy
RAPN: Robot-assisted partial nephrectomy
CT: Computed tomography
